# Wound healing potential of Spirulina platensis extracts on human dermal fibroblast cells

**DOI:** 10.17179/excli2014-697

**Published:** 2015-03-02

**Authors:** Pauzi Nur Aimi Syarina, Govindarajan Karthivashan, Faridah Abas, Palanisamy Arulselvan, Sharida Fakurazi

**Affiliations:** 1 Laboratory of Vaccines and Immunotherapeutics, Institute of Bioscience, Universiti Putra Malaysia, 43400 UPM Serdang, Selangor, Malaysia; 2 Department of Food Science, Faculty of Food Science and Technology, Universiti Putra Malaysia, 43400 UPM Serdang, Selangor, Malaysia; 3 Department of Human Anatomy, Faculty of Medicine and Health Sciences, Universiti Putra Malaysia, 43400 UPM Serdang, Selangor, Malaysia

**Keywords:** Spirulina platensis, wound healing activity, aqueous extract, LC-MS/MS, wound scratch assay

## Abstract

Blue-green alga (*Spirulina platensis*) is a well renowned nutri-supplement due to its high nutritional and medicinal properties. The aim of this study was to examine the wound healing efficiency of *Spirulina platensis* at various solvent extracts using *in vitro* scratch assay on human dermal fibroblast cells (HDF). Various gradient solvent extracts (50 μg/ml of methanolic, ethanolic and aqueous extracts) from *Spirulina platensis* were treated on HDF cells to acquire its wound healing properties through scratch assay and in this investigation we have used allantoin, as a positive control to compare efficacy among the phytoextracts. Interestingly, aqueous extract were found to stimulate proliferation and migration of HDF cells at given concentrations and enhanced closure rate of wound area within 24 hours after treatment. Methanolic and ethanolic extracts have shown proliferative effect, however these extracts did not aid in the migration and closure of wound area when compared to aqueous extract. Based on phytochemical profile of the plant extracts analyzed by LC-MS/MS, it was shown that compounds supposedly involved in accelerating wound healing are cinnamic acid, narigenin, kaempferol, temsirolimus, phosphatidylserine isomeric derivatives and sulphoquinovosyl diacylglycerol. Our findings concluded that blue-green algae may pose potential biomedical application to treat various chronic wounds especially in diabetes mellitus patients.

## Introduction

Wound healing is a dynamic process involving complex interactions between cellular, molecular, biochemical and physiological activities which result in the regeneration and replacement of injured connective tissue at the wound site (Velnar et al., 2009[29]). Normal wound undergoes series of event that involve in wound healing process. Initial stages of wound healing engaged the formation of a blood clot and inflammation, which occur immediately upon injury. Following 4 days injury, the inflammatory response is followed by proliferation and migration of dermal and epidermal cells, and matrix synthesis, in order to fill the wound gap and re-establish skin barrier (Hackam and Ford, 2002[10]; Werner et al., 2007[31]). Finally, tissue remodeling and maturation enable full recovery of the skin tissue which sometimes leaving no remaining trace of the previous skin damage (Diegelmann and Evans, 2004[8]; Harding et al., 2005[11]). 

The increasing popularity of natural therapy has led podiatrist hope of finding a more effective and cost effective product for healing of chronic wounds (Ananda, 2012[2]). Since, ancient years people have been using plants as treatments to accelerate wound healing process though without any scientific evidence of the plant efficiency and very little knowledge about the compounds and mode of actions. Plants are believed to have natural therapeutic effects against endocrine disorders and other diseases (Arulselvan et al., 2014[3]) and can be treated for any kind of injury such as cut or burned, with simple formulation procedures to make them the best candidate as natural remedies. 

Algae have expanded growing interest in their values as medicine and functional foods (Barrow and Shahidi, 2007[4]) as they provide harmless and nutritious product for healthcare supplement market (Dominguez, 2013[9]). *Spirulina platensis* is a filamentous blue-green alga originates in many alkaline lakes with a high pH. It contains approximately 70 % easily digestible protein where 18 out of 22 amino acids and all of the essential amino acids are available, making it a unique vegetarian source of complete protein (Somchit et al., 2007[27]). 

Since, wound healing is a complex biological process, there are a few methods of *in vitro* and *in vivo* assays are available to study the first insight of how plant preparation can possibly influence the regeneration of new tissue and enhance the wound. However, among all the methods present, *in vitro* scratch assay has been proven a valuable and inexpensive tool to study wound healing potential of drugs (Liang et al., 2007[15]). 

The objective of present study was to evaluate the efficacy of different extracts of *Spirulina platensis *in accelerating the proliferation and migration into the monolayer of human dermal fibroblast cells (HDF) and to determine the bioactive compounds by secondary metabolite profiling responsible for wound healing potential.

## Materials and Methods

### Preparation of extract

The *Spirulina platensis (*SP*)* were purchased from Algaetech International Sdn. Bhd. The *Spirulina platensis* extract were prepared by maceration of 10 g of freeze-dried powder in 500 ml of different solvents (methanol, ethanol and ultrapure water) for 24 h at room temperature. The mixture was then centrifuged at 3000 rpm for 10 min (4 °C) and the supernatant was filtered (Whatman No. 1) to remove cell debris. All samples were then evaporated at 40 °C by vacuum rotary evaporator. The dried extracts from each solvent were stored at 4 °C until further experiments.

### Cell culture and plant extract preparation for treatment 

Normal Adult Human Primary Dermal Fibroblast cell (HDF) was purchased from ATCC (American Type Culture Collection, USA). Cells were maintained in complete DMEM high glucose media with 5 % FBS and 1 % of penicillin-streptomycin in a humidified 5 % CO_2_ incubator at 37ºC.

Methanolic, ethanolic and aqueous extracts solution were freshly prepared for each experiment by dissolving each dried sample in cell culture medium and sterilized by syringe filter (0.22 µm). Concentration used in the experiment was based on dry weight of the extract (mg/ml).

### Cytotoxicity assay

Cytotoxicity assay was conducted to determine the range of concentrations of extract to be used for scratch assay and further *in vitro* analysis. HDF cells were cultured in 96 well culture plates at density 1 × 10^6 ^cells/ml with DMEM complete media for 24 h. The medium was replaced with 100 µl of methanolic, ethanolic and aqueous extract solution with different concentration (50, 100, 150, 200, 250, 300 µg/ml) for 24 h. The cell viability was assessed using 3-[4,5-Dimethylthiazol-2-yl]-2,5-diphenyltetrazolium bromide (MTT) solution and MTT powder was dissolved in phosphate-buffered saline (PBS) at a concentration of 5 mg/ml. MTT was added to each well (20 μl) and plates were incubated at 37 °C for 3 hrs. The medium was replaced with 100 μl DMSO and the absorbance for each well was measured at 570 nm on a microplate reader.

### In vitro scratch assay 

HDF cells were seeded in 24-well plates at a density of 3×10^6^ cells/ml and allowed to grow for 24 h at 37 °C and 5 % CO_2_. A small linear scratch was created in the confluent monolayer by gently scraping with sterile p200 pipette tips (care was taken during scratching process to ensure universal size and distant was made for all samples). Cells were extensively rinsed with PBS to remove cellular debris before adding the media with different treatment solution (methanolic, ethanolic and aqueous extracts) at concentration of 50 µg/ml. Allantoin (Sigma Aldrich, Germany), was used as a positive control drug and cells without treatment was used as negative control. After 24 h, images of migrated cells were taken using digital camera connected to inverted microscope to observe the closure of wound area. All scratch assays were performed in quadruplicate.

### LCMS/MS analysis

Mass spectra were acquired using AB Sciex 3200 QTrap LCMS/MS with Perkin Elmer FX 15 UHPLC system (MA, USA). The negative ion mass spectra were obtained from the LC QTrap MS/MS detector on full ion scan mode (100 to 1200 m/z for full scan and 50-1200 m/z for MS/MS scan) at a scan rate of 0.5 Hz. The hyphenated system was supported with a mass spectrometry software and spectral library provided by ACD labs (TO, CA). Analyte separation was carried out on a pre-packed C18 (4 × 250 mm, 5 μm, Phenomenex) column with a gradient mobile phase comprising water (solvent A) and methanol with 1 % acetonitrile (solvent B), each containing 0.1 % formic acid and 5mM ammonium formate. The gradient program commenced with 80 % to 90 % solvent B from 0.01 to 11.00 min with a flow rate of 1.0 ml/min. The injection volume was set to 20 µL. All chromatographic procedures were performed at ambient temperature and the corresponding peaks from QTrap LCMS/MS analysis of the aqueous extracts of *Spirulina platensis *were identified by comparison with the literature data / ACD labs mass spectral library.

### Statistical analysis

All experimental values are presented as mean ± SD. The data were analyzed using ANOVA, and then paired t-test was used to analyze the difference between the groups. P-values at less than 0.05 were considered as statistically significant.

## Results

### Effect of Spirulina platensis extracts on HDF cells viability 

**Figure 1:**
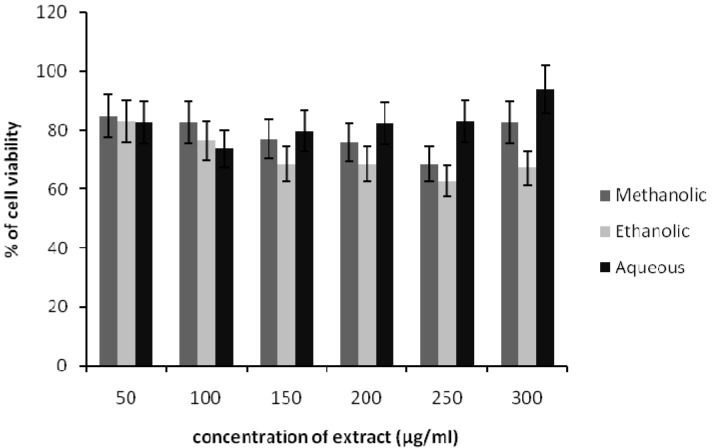
Effects of methanolic, ethanolic and aqueous extracts of *Spirulina platensis* on the viability of HDF cells. The cells were incubated for 24 h before being assessed for their viability. Data presented as mean ± SD.

The viability of HDF cells was reduced by the treatment of methanolic, ethanolic and aqueous Spirulina extract up to 300 μg/ml (Figure 1(Fig. 1)). The concentration of ethanolic extract at 150 - 300 μg/ml showed 30-40 % cyto-toxicity after 24 h incubation. At a dose of 50 μg/ml of each extract, the HDF cells have shown more than 80 % of cell viability. Further, increase in extract concentration led to gradual decrease in cell viability as higher concentrations were found to be cytotoxic to the cells. Therefore, the non-toxic concentration of extract was used for further testing in scratch assay. 

### Effect of Spirulina platensis extracts on in vitro scratch assay

**Figure 2:**
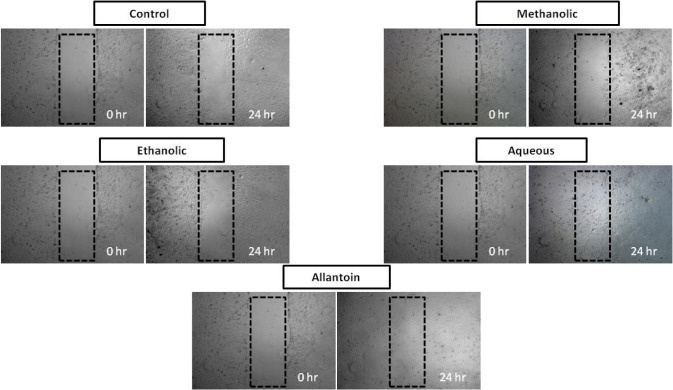
*In vitro* scratch assay (x4 magnification). HDF cells were injured and cell migration assay with and without treatment was performed. Aqueous extract treated fibroblasts showed faster cell proliferation and migration as compared with those untreated cells as well as those cells treated with methanolic and ethanolic extract.

Figure 2(Fig. 2) shows the images of scratch assays on HDF cells at 0 and 24 h post injury time without treatment (control) and with treatment. All the images are shown progression of wound closure on scratch wounded HDF cells. Enhanced migration and wound closure were observed in aqueous extract treated cells as compared with those of untreated cells in which the degree of cell migration and cell proliferation are slow. Rapid cell migration and wound closure rate of aqueous extract treated HDF cells were observed and these effects were comparable with positive control drug, allantoin. Wound area at 24 h post injury remained open for methanolic and ethanolic *Spirulina* extract on scratch wounded HDF cells.

### LCMS/MS analysis of Spirulina platensis extracts

**Figure 3:**
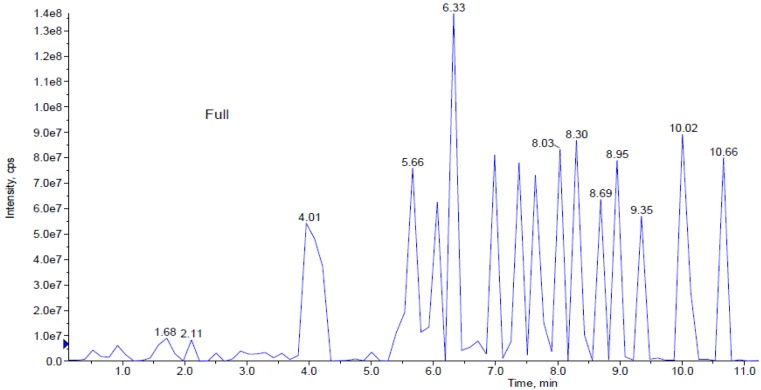
This figure represents peaks extracted from the aqueous extract from 0.0 to 11.0 min. Further analysis of LCMS/MS QTrap with an adaptive data dependent gradient program, it was revealed to be phenolic compounds and fatty acid derivatives.

Based on *in-vitro* scratch assay which used methanol, ethanol and aqueous extracts of *S. platensis* for treatment, it was found that aqueous extract has shown significant wound healing activity on HDF cells. Further analysis of *S. platensis* aqueous extract through LCMS/MS QTrap with an adaptive data dependent gradient program, the compounds were revealed to be phenolic compounds and fatty acid derivatives (Figure 3(Fig. 3)). Tentatively, few compounds have been identified and enlisted in Table 1(Tab. 1) based on the literature data and Advanced Chemistry Development (ACD) labs based mass spectral library.

Peak 1 which was released at 0.93 min was identified as cinnamic acid based on its parent ion of m/z 147 (Wang et al., 2008[30]). Peak 2 with m/z 271 was identified as narigenin (Sanchez-Rabaneda et al., 2003[24]), peak 3 gave molecular ion peak at m/z 285 was recognized as kaempferol (Sanchez-Rabaneda et al., 2003[24]) whereas peak 4 was found to be temsirolimus express significant parent ions at m/z 1046 (Cai et al., 2007[6]). Peaks 5-12 which were released between 5.6 to 8.95 min were identified as phosphatidylserine isomeric derivatives which revealed a same parent ion at m/z 834 and 835 as well as the same MS^2 ^fragment ions at m/z 554, 279, 391, 255 and 241, which made them as isomers (Tyurin et al., 2008[28]). Peaks 14 and 15 produced molecular ion peak at m/z 817 and MS^2^ fragment ions at m/z 537, 255 as well as 225. Based on this significant fragmentation pattern and comparison with previously reported data, the peaks 14 and 15 were found to be sulphoquinovosyl diacylglycerol (SQDG) (Mizushina et al., 2003[19]).

In the present study, tentative active compounds such as hydroxylated cinnamic acid, narigenin, kaempferol, phosphatidylserine isomeric derivatives and sulphoquinovosyl diacylglycerol (SQDG) have identified from *S. platensis* aqueous extract by LC-MS/MS analysis. Until now, the most general compounds that have been reported in *S. platensis* extract are phenolic compound, carotenoids, phycobiliproteins, chlorophyll, polyunsaturated fatty acids, sulphated polysaccharides and sterols (Herrero et al., 2007[13]).

## Discussion

The skin provides protection and acts as external barrier of body cells and tissues against microbial infection from external environment. Any damage to skin barrier must be rapidly and effectively restored via wound healing process. Dermal fibroblast is the first line defense which responses to injury and is essential for cutaneous wound repair by proliferation and migration process into the wound site. It differentiates into myofibroblasts as a response to macrophage-derived cytokines such as TGF-β1, which is reliant on their contact with fibronectin (Serini et al., 1998[26]). Proliferation and migration of the cells are important events in wound healing process, therefore, the study of natural products and their active compounds which influence the migration of fibroblast may help in improving the cutaneous wound healing progression.

To make sure that effect of extract treatment on HDF proliferation and migration were not interfere by any toxicity; we determined the viability of fibroblast after 24 hours in different extracts concentrations. Based on cell viability assay, our results demonstrated that low concentration of methanolic, ethanolic and aqueous extract did not give any cytotoxicity effects on HDF cells after 24 hours of treatment. These findings indicated that 50ug/ml was non-toxic concentration of *Spirulina platensis* crude extracts and this concentration was used for further treatment. 

Scratch assay is a suitable and inexpensive screening method to differentiate and verify natural products for their *in vitro* wound healing activity. This assay is highly related to the second phase of wound healing process characterized by proliferation and migration of the keratinocytes and fibroblasts (Schafer and Werner, 2007[25]; Liu et al., 2013[16]). In this study, the crude extracts from *Spirulina platensis* were used as treatment during scratch assay, aqueous extract increases the population of HDF cells in the 'wounded' or scratched area due the migration of cells and also by proliferation of the migrated cells. Conversely, methanolic and ethanolic extracts from *Spirulina platensis* showed some increment in the abundance cells yet they did not aid in cell migration. This may explain the non-healing activity of HDF cells when treated with those extracts. Our results are in agreement with that of a previous study which explained the wound healing potential of methanolic extract of *Moringa oleifera* leaves against stimulated HDF cells based on its wound closure activity (Muhammad et al., 2013[21]). 

In the present study, LC-MS/MS analysis on *S. platensis* aqueous extract compounds revealed as hydroxylated cinnamic acid, narigenin, kaempferol, phosphatidylserine isomeric derivatives and sulphoquinovosyl diacylglycerol (SQDG) compounds. Until now, the most general compounds that have been reported in *S. platensis* extract are phenolic compound, carotenoids, phycobiliproteins, chlorophyll, polyunsaturated fatty acids, sulphated polysaccharides and sterols (Chojnacka et al., 2012[7]; Lordan et al., 2011[18]; Pumas et al., 2011[22]). It has been previously reported that hydroxyl cinnamic acid and their derivatives inhibit diabetic complications mediated by advanced glycation end products formation and moderately induce corneal epithelial wound healing by inhibiting protein tyrosine phosphatases (PTP)-1β gene (Adisakwattana et al., 2013[1]). Narigenin is an effective inhibitor of the pro-inflammatory cytokine (IL-1β, IL-6, IL-8 and TNF-α) response stimulated by lipopolysaccharide in both macrophages and in whole blood (Bodet et al., 2008[5]). Kaempferol has been reported to possess anti-oxidant (Karthivashan et al., 2013[14]) and anti-inflammatory activity via its inhibition of aldosterone signaling and aldosterone-induced gene expression in HUVECs (Liu et al., 2000[17]). Ramstrom et al. in (2003[23]) reported that phosphatidylserine plays an essential role in wound healing as a pro-coagulant in platelet formation process. It has also been reported that collagen-adherent platelets activate blood coagulation by exposing phosphatidylserine (PS) at wound site (Heemskerk et al., 2002[12]). In general, fatty acid and its derivatives promote wound healing by angiogenic activity, Mizushina et al. (2003[20]) reported that sulphoquinovosyl diacylglycerol (SQDG) as a selective inhibitor of eukaryotic DNA polymerases a and b and also an immunosuppressive agent. These reports strongly suggest that bioactive compounds such as, hydroxylated cinnamic acid, phosphatidylserine isomeric derivatives, sulphoquinovosyl diacylglycerol (SQDG), present in *S. platensis *aqueous extract are virtuously responsible for its enhanced wound healing activity.

## Conclusion

Our findings suggested that *Spirulina platensis*, aqueous extract showed highest wound healing activity and might be considered as a potential source of therapeutic agent for chronic wound and its associated complications. In addition, this study demonstrated that scratch assay is a suitable and economical method that gives dependable results for the proliferation as well as migration of the HDF cells in an artificial wounded model. However, further study need to be conducted to confirm their biological activity more specially in experimentally induced diabetic wound model. Further, isolation, identification and purification active compounds those are possess wound healing potential are in progress in our research group.

## Acknowledgements

This research work was supported by Research University Grant Scheme (RUGS) of Universiti Putra Malaysia (05-02-11-1419RU and 04-02-12-2089RU).

## Conflict of interest

The authors have declared no conflict of interest.

## Figures and Tables

**Table 1:**
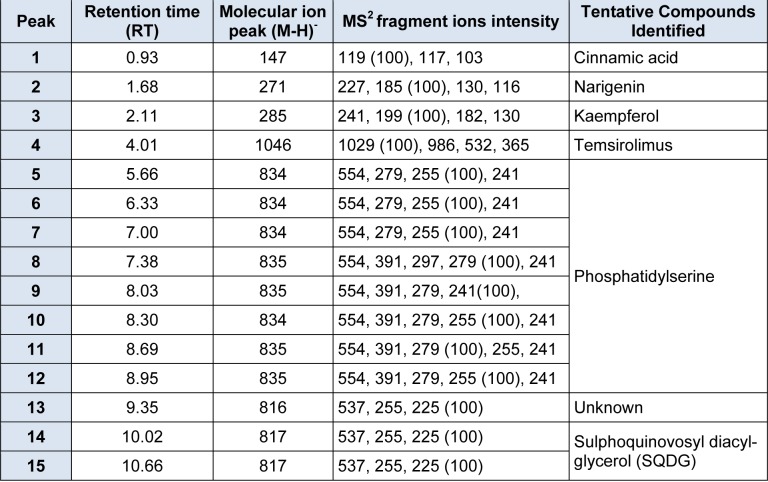
Retention times, MS and MS^2^values of the major bioactive constituents present in *S. platensis* aqueous extract by HPLC-DAD-ESI-MS/MS.
